# Prolonged outpatient parenteral antimicrobial treatment: frequency and evolution over a six-year period in a Swiss University Hospital

**DOI:** 10.1186/s12879-024-10170-0

**Published:** 2024-11-07

**Authors:** Aline Munting, José Damas, Lyne Arensdorff, Matthias Cavassini, Serge de Vallière

**Affiliations:** 1 Infectious Diseases Service, University Hospital of Lausanne and University of Lausanne, Lausanne, Switzerland; 2 Center for Primary Care and Public Health (Unisanté), University of Lausanne, Lausanne, Switzerland

**Keywords:** OPAT, Parenteral antibiotic therapy, Infectious diseases, Prolonged IV antibiotics

## Abstract

**Background:**

Emerging research indicates the potential for early transition from intravenous to oral antimicrobial therapy in certain infections. This trend may have implications for outpatient parenteral antibiotic therapy (OPAT) programs, as the demand for prolonged intravenous treatment could decrease. The objective of this study was to evaluate the frequency and evolution of OPAT courses of ≥ 14 days over the years and determine the medical justification for those prolonged treatments.

**Methods:**

All patients treated intravenously for ≥ 14 days by the OPAT program at Lausanne University Hospital, Switzerland, between 2017 and 2022 were included in the study. Data were extracted from a prospectively established OPAT database. Prevalence of prolonged antibiotic treatment and its clinical and microbiological information were identified.

**Results:**

During the study period, a total of 2,448 treatment courses were administered: 1,636 intravenous (IV) and 812 oral treatments. Of the IV treatments courses, 749 (36%) were of a duration of ≥ 14 days, without discernible trend over the 6-year study period. The most common type of infections needing prolonged treatment were bone and joint infections (31%), endovascular infections (18%), complicated intra-abdominal infections (15%), and urinary tract infections (11%), with only minor fluctuations in these proportions during the study period. Finally, the use of second-line antibiotics (piperacillin-tazobactam, carbapenems and vancomycin) did not increase over the years, suggesting that prolonged intravenous antibiotic therapy is not linked to an increase of anti-microbial resistance in our cohort.

**Conclusions:**

Despite the general trend towards shorter intravenous treatment courses in infectious diseases, our OPAT unit did not observe a decline in the use of prolonged intravenous antibiotic therapy between 2017 and 2022, suggesting that OPAT units will probably not see a decrease in their activities in the near future.

**Supplementary Information:**

The online version contains supplementary material available at 10.1186/s12879-024-10170-0.

## Introduction

Outpatient parenteral antimicrobial therapy (OPAT) is an established approach for treating patients with challenging infections who do not require continued hospitalization [1–3]. In recent years, several studies have shown that oral antimicrobial treatment can often achieve outcomes equivalent to intravenous therapy for certain types of infections. Notably, this has been observed in specific patient subgroups with bone and joint infections or endocarditis, which have traditionally required several weeks of intravenous treatment [4, 5]. These findings could have a significant impact on the future activity of OPAT programs as prolonged intravenous treatments might become less commonly prescribed. To effectively plan for these programs, it is therefore crucial to understand if those results are already translating into a reduction in the use of prolonged intravenous treatments.

The objective of this study was to evaluate the evolution of prolonged intravenous antimicrobial treatments (≥ 14 days of duration), administered at our OPAT unit and to determine the medical justification for these long treatments.

## Methods

### Structure and organisation of OPAT unit

Lausanne University Hospital in Switzerland established an OPAT program in 2014, integrated within the infectious diseases department. After a successful three-year implementation period with a progressive increase in the number of referred patients, the unit has been caring for 300 to 350 patients annually since 2017. The service currently includes four full-time nursing positions and one full-time physician resident, supervised by an infectious disease specialist. They define the treatment plan with the in-hospital teams and make sure that the situation is compatible with outpatient care. Antibiotic administration is available through three models: daily infusions at the OPAT unit, self-administration at home, or administration at home by a visiting nurse. Self-administration is largely the most used approach (> 60% of the OPAT patients). Antimicrobial treatments are administered by intermittent or continuous infusions. Elastomeric pumps are used for continuous infusion when antibiotics are considered stable in these devices according to the literature. Upon entry into the OPAT service, close clinical follow-up is ensured with patients seen at least once a week for clinical and laboratory evaluations. Referrals to the OPAT unit come from all hospital services, almost systematically following a consultation by an in-house infectious disease specialist, who provides recommendations for both inpatient treatment and outpatient care, based on international guidelines and local epidemiology. All patient treatments are reviewed weekly by the OPAT infectious diseases team to monitor progress under antibiotic therapy, assess potential side effects, reevaluate treatment duration and evaluate the possibility of a switch to oral treatment. Of note, patients who receive an early switch to oral therapy during hospitalization or upon discharge are not followed by the OPAT service.

### OPAT database

The OPAT unit established in 2014 a prospective registry (ClinicalTrials.gov identifier NCT 03221140, registration date 01.01.2014) to assess the efficacy and safety of the treatments administered. This study received approval from the Ethics Committee of the Canton of Vaud, Switzerland (protocol ID 34/14). A research assistant from the OPAT unit continually enters the data into a secuTrial® database. The database includes the following information: demographics of the patients (age and sex), types of infections, antimicrobial treatments, side effects, non-infectious complications, and treatment outcomes (including cure, treatment failure, and rehospitalization) at three months after the end of OPAT treatments. In 2021, we also added the treatment goal, which can be categorized as “cure”, “treatment until new surgical intervention”, or “stabilization of incurable infection”.

### Study cohort

In this single center, prospective cohort study, we included all patients who provided informed consent to participate in the registry and had received ≥ 14 days of parenteral treatment at our OPAT unit between 2017 and 2022. We opted not to include patients treated prior to 2017 due to the run-in period of the OPAT unit’s activity. Furthermore, the period between 2017 and 2022 was chosen as several studies conducted during this time indicated that early transitioning from intravenous to oral treatment could be feasible for certain infections, leading to comparable outcomes and fewer complications.

We extracted for each subject the following information from the registry: demographic information, infection diagnosis, treatment goal, type and duration of antimicrobial therapy, and outcome assessed three months after completing treatment. We determined the justification for prolonged intravenous treatment based on the type of infection and the identification of the microorganism involved.

### Statistics

All analyzes were descriptive. We presented continuous variables as median and interquartile ranges. We registered categorical variables as counts and percentages. Categorical variables were compared with the chi-square test or Fisher’s exact test, and continuous variables were compared with the Wilcoxon rank-sum test.

## Results

**Fig. 1:**
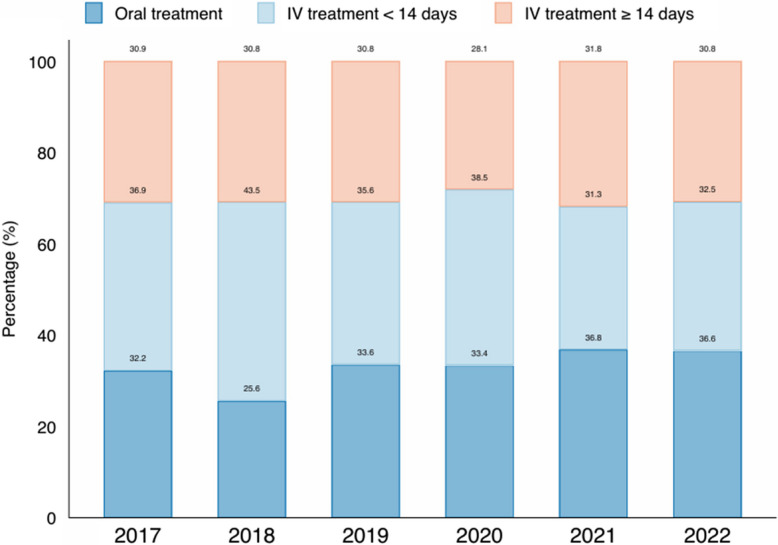
Proportion of oral and IV treatments at the OPAT unit during the study period. The Y axis shows the percentage of patients followed by the OPAT unit and receiving oral antibiotic treatments (dark blue), IV antibiotic treatments of < 14 days (light blue) and IV antibiotic treatments of ≥ 14 days (red). The X axis represents each year across the study period

**Fig. 2:**
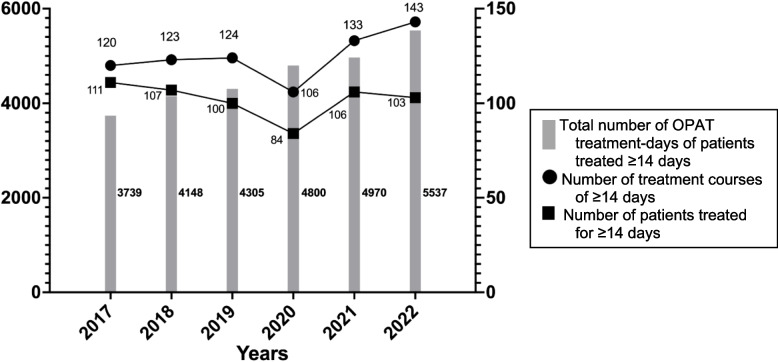
OPAT of ≥ 14 days. The left Y axis shows the total cumulative number of treatment-days of patients receiving ≥ 14 days of intravenous antimicrobial treatment, and it corresponds to the columns. The right Y axis shows the number of treatment courses and number of patients receiving ≥ 14 days of intravenous antimicrobial treatment, corresponding to the connected lines. The X axis represents each year across the study period. OPAT: outpatient parenteral antimicrobial therapy

Between 2017 and 2022, the OPAT unit administered a total of a total of 2,448 treatment courses: 1,636 intravenous and 812 oral treatments. Of the IV treatments courses, 749 were administered for ≥ 14 days. The proportion of intravenous treatment courses of ≥ 14 days remained stable through the study period, compared to oral antibiotic treatments followed by OPAT unit (Fig. 1). The number of OPAT treatment days increased from 3,739 to 5,537 days (Fig. 2). The patients of our study population had a median age of 59 years (range: 21 to 95 years) and 29% were females.

**Fig. 3:**
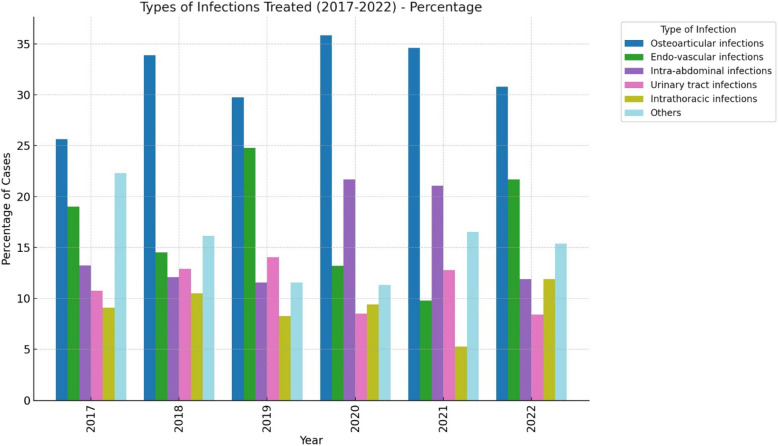
Types of infections treated for ≥ 14 days by OPAT unit. * Others include intra-cerebral infections (37), primary bacteraemia (13), catheter infections (23), Ear nose and throat (19), muco-cutaneous (21) and gynaecologic infections (4)

Figure 3 and Table S1 summarize the types of infections treated with long duration antibiotic therapies. The most common infections were bone and joint infections, accounting for 31% of cases. This was followed by endovascular infections (18%), complicated intra-abdominal infections (15%), and urinary tract infections (11%). The most common bacteria treated were *Staphylococcus aureus* (22.3%), *Escherichia coli* (17%), *Pseudomonas aeruginosa* (12%), *Klebsiella pneumoniae* (9%), *Enterococcus faecalis* (9%) and *Enterococcus faecium* (9%).

The treatment goal was included in the registry starting from 2021, resulting in missing information for 70% of the treatment courses. Among the treatment courses with available information, 83% aimed for “cure”, while 16% aimed for “stabilization before new surgical treatment” or “stabilization of an uncurable infection”.

**Fig. 4:**
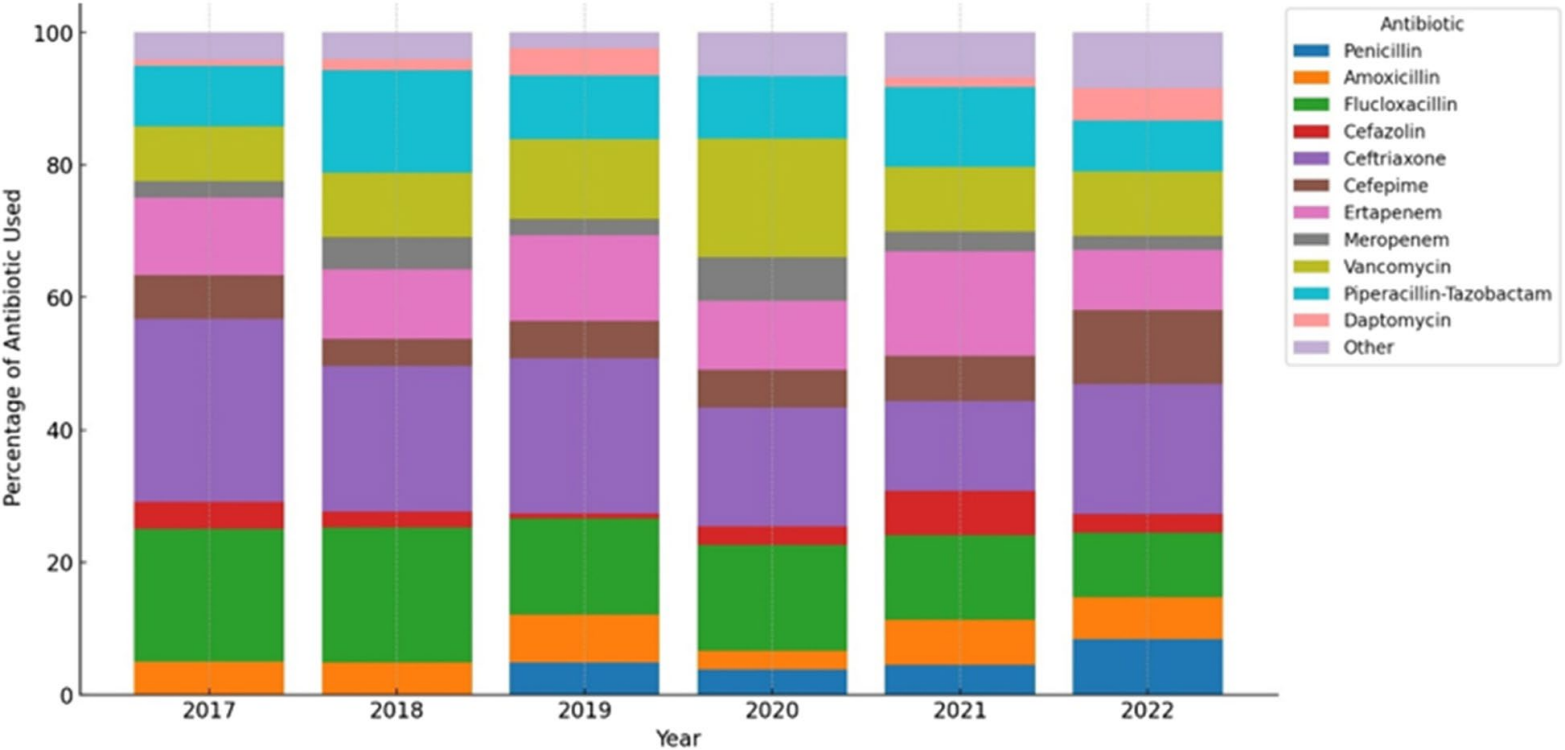
Antibiotics used for OPAT of ≥ 14 days

Figure 4 and Table S2 shows the type of antibiotics used for ≥ 14 days. Ceftriaxone was the most commonly used antibiotic, accounting for 21% of cases, followed by flucloxacillin (15%) and ertapenem (12%). The use of piperacillin-tazobactam, cefepime, carbapenems (ertapenem and meropenem) and vancomycin remained stable throughout the study period.

The median duration of OPAT was 23 days, with an interquartile range of 17 to 33 days, with no differences between sex. The main reason for stopping OPAT was end of treatment as planned for 58% and switch to oral therapy in 23% of patients (Table 1). Antibiotic therapy was discontinued in two percent of patients to create a “therapeutic window”, meaning treatment was stopped before a planned surgical reintervention to obtain microbiological samples without antibiotic pressure. At the three-month follow-up, 3.6% of the included patients had died. Out of those patients, 82% (18 out of 22) were in palliative situation with an expected prognosis of death within months. 73% were cured, 20% experienced treatment failure, and 7% had missing data.

**Table 1:** Reasons to stop OPAT of ≥ 14 days

Reasons to stop intravenous antibiotic treatment	Number of episodes	%
End of treatment	434	58
Switch to oral therapy	172	23
Hospitalisation (planned and unplanned)	96	13
Change of intravenous treatment	15	2
Side effects	18	2
Therapeutic window	14	2
**Total**	749	100

## Discussion

Prolonged intravenous antimicrobial therapy can impose significant burdens on patients compared to oral treatment, impacting autonomy and increasing complications, particularly from IV catheters. Additionally, IV therapy is more costly. As a result, there is growing interest in exploring the possibility of an early switch from intravenous to oral antimicrobial therapy.

Several studies have demonstrated that oral treatments with good bioavailability can be non-inferior to parenteral therapies [6–8]. Two landmark studies published in 2019 showed the safety of early oral transitions for select patients with endocarditis and bone and joint infections, traditionally managed through prolonged intravenous treatment courses. The POET (Partial Oral Treatment of Endocarditis) trial showed that patients with certain types of endocarditis could safely switch to oral antibiotics after at least 10 days of IV therapy [4]. The OVIVA (Oral versus intravenous antibiotics for bone and joint infections) study also demonstrated the non-inferiority of early transition from intravenous to oral antibiotic therapy within one week for osteoarticular infections [5].

These findings suggest that OPAT units may experience a significant decrease in activity in the future, as intravenous treatments of long duration might become less commonly prescribed. However, this study conducted at our OPAT unit showed that both the absolute number and the proportion of patients receiving intravenous antimicrobial therapy for ≥ 14 days have not decreased over the past six years. Among our cohort, the most common conditions requiring prolonged intravenous treatment were bone and joint infections (31%), endovascular infections (18%), complicated intra-abdominal infections (15%), and urinary tract infections caused by ESBL-producing Enterobacterales (11%).

The stability of prescription of second-line antimicrobials such as piperacillin-tazobactam, carbapenems and vancomycin throughout the study period suggests that rising resistance is not a primary factor for the continued use of extended IV therapy.

Our internal treatment guidelines for prosthetic joint infections recommended already before the results of OVIVA trial to consider an oral switch after 14 days. These recommendations are followed for most patients unless no oral option is available. Prolonged IV therapy for osteo-articular infections was primarily due to resistance to fluoroquinolones in 20% of cases (data not shown) and logistical issues related to prosthetic re-implantation surgeries.

Infective endocarditis has traditionally been treated by 4 to 6 weeks of intravenous antibiotics [9, 10]. A meta-analysis published in 2020 which included 21 observational studies and 3 randomized controlled trials suggested that oral treatment is non-inferior to intravenous treatment in selected cases [11]. The authors concluded that this is rather good evidence that an oral antibiotic therapy is a reasonable approach for patients who meet the following criteria: clinically stable with no immediate indication for cardiac surgical intervention; cleared bacteremia; absence of concerns regarding absorption or adherence to oral therapy; and finally if an oral antibiotic regimen is available [11]. Nonetheless, our data show that the number of endovascular infections and more specifically infective endocarditis treated with ≥ 14 days of intravenous treatment has not decreased over the six-year study period in our OPAT unit.

We believe that several factors are responsible for this absence of change in the treatment of infective endocarditis. The heterogeneity of oral step-down regimens in the different trials and the limited number of eligible subjects in most studies, are probably the main factors. Existing data do not provide evidence for a clear duration of the intravenous lead-in before the switch to oral dual-combination therapy. Moreover, the oral step-down therapy was done in most studies under very specific conditions. For example, oral treatment was introduced in the POET trial only after a repeat endo-oesophageal echocardiography. Because of these stringent inclusion criteria, only 20% of the patients screened were finally switched to oral treatment in the POET trial. The recommendation of an endo-oesophageal echocardiography before oral switch has also been included in the recently published endocarditis guidelines of the European Society of Cardiology [12]. This recommendation will probably delay the decrease of prolonged intravenous treatment for infective endocarditis, as a control of endo-oesophageal echocardiography is rarely done in our center. Finally, it cannot be denied that there is a probable reluctance of physicians to move away from the intravenous administration of antibiotics for a severe disease such as infective endocarditis.

Regarding urinary tract infections, 65% (55/84) of cases treated for over 14 days were infections caused by ESBL-producing Enterobacterales. While oral fosfomycin is effective for cystitis caused by ESBL-producing *E. coli*, it is not recommended for pyelonephritis, despite evidence suggesting it could be a effective alternative [13, 14].

A notable portion in our cohort was represented by patients with uncurable infections. Most of these patients had severe underlying conditions precluding surgical treatment, which would otherwise have been required for definitive treatment. With intravenous treatment, many of these infections could still be controlled and allowed these patients to return home for long periods of time.

Limitations of our study include a lack of data on the duration of in-hospital IV therapy before outpatient treatment, which may lead to underestimating the total IV treatment duration. Additionally, an early switch to oral antibiotics when patient is discharged home was overlooked as our database captured only the proportion of oral treatments when prior IV treatments were administered with OPAT. Furthermore, patient comorbidities and comprehensive antibiotic susceptibility results are not collected in the database; however, the stable use of second-line antibiotics suggests no significant changes in resistance patterns.

In conclusion, prolonged parenteral antimicrobial therapies are prescribed to a significant number of patients managed by our OPAT unit, with no trend towards a decrease of these prolonged treatments.

## Supplementary Information

Supplementary Material 1: Supplementary appendix. Table S1. Types of infections treated for ≥ 14 days by OPAT unit. Table S2. Antibiotics used for OPAT of ≥ 14 days.

## Data Availability

The datasets generated and analyzed for this study are available from the corresponding author after publication upon reasonable request.

## Abbreviations

OPAT: Outpatient parenteral antibiotic therapy
IV: Intravenous
POET: Partial Oral Treatment of Endocarditis
OVIVA: Oral versus intravenous antibiotics for bone and joint infections
E. coli: Escherischia coli
