# Correction: Prolonged outpatient parenteral antimicrobial treatment: frequency and evolution over a six-year period in a Swiss university hospital

**DOI:** 10.1186/s12879-025-11041-y

**Published:** 2025-05-05

**Authors:** Aline Munting, José Damas, Lyne Arensdorff, Matthias Cavassini, Serge de Vallière

**Affiliations:** 1 Infectious Diseases Service, University Hospital of Lausanne and University of Lausanne, Lausanne, Switzerland; 2 Center for Primary Care and Public Health (Unisanté), University of Lausanne, Lausanne, Switzerland

**Correction: BMC Infectious Diseases**

10.1186/s12879-024-10170-0

Following publication of the original article [1], we were notified that the authors’ first and last names were incorrectly tagged.

Originally published names: Munting A., Damas J., Arensdorf L., Cavassini M. and de Vallière S.

Corrected names: Aline Munting, José Damas, Lyne Arensdorff, Matthias Cavassini, Serge de Vallière

The original article has been corrected.

## References

[1] Munting, et al. BMC Infect Dis. 2024;24:1255. 10.1186/s12879-024-10170-0 10.1186/s12879-024-10170-0 PMC11542217 39511471

