# Supplementary material for: Prolonged outpatient parenteral antimicrobial treatment: frequency and evolution over a six-year period in a Swiss University Hospital
Source: BMC Infect Dis. 2024 Nov 7;24:1255. doi: 10.1186/s12879-024-10170-0 (PMC11542217; doi:10.1186/s12879-024-10170-0)
Supplement: Supplementary file 1 — Supplementary Material 1: Supplementary appendix. Table S1. Types of infections treated for ≥ 14 days by OPAT unit. Table S2. Antibiotics used for OPAT of ≥ 14 days. [file 12879_2024_10170_MOESM1_ESM.docx]

**Supplementary appendix**

**Table 1. Types of infections treated for** ≥**14 days by OPAT unit**

| **Types of infection** |  |  |  |  |  |  |  |
| --- | --- | --- | --- | --- | --- | --- | --- |
|  | **2017** | **2018** | **2019** | **2020** | **2021** | **2022** | **Total** |
| **Osteoarticular infections** | **31**  **(13%)** | **42**  **(18%)** | **36**  **(15%)** | **38**  **(16%)** | **46**  **(19%)** | **44 (19%)** | **237** |
| - Osteomyelitis | 9  (12%) | 12  (16%) | 11 (15%) | 12  (16%) | 12 (16%) | 19 (25%) | 75 |
| - Spondylodiscitis | 5  (19%) | 4  (15%) | 3  (12%) | 3  (12%) | 6  (23%) | 5  (19%) | 26 |
| - Sternitis | 0 | 0 | 2 (100%) | 0 | 0 | 0 | 2 |
| - Septic arthritis | 3  (12%) | 11  (42%) | 2  (8%) | 4  (15%) | 3  (12%) | 3  (12%) | 24 |
| - Prosthetic joint infections | 8  (13%) | 3  (5%) | 15 (24%) | 12  (19%) | 17 (27%) | 8  (13%) | 63 |
| - Other implant associated infections | 5  (9%) | 12  (21%) | 7  (13%) | 7  (13%) | 11 (20%) | 14 (25%) | 56 |
| - Other | 2  (15%) | 4  (31%) | 1  (8%) | 2  (15%) | 2  (15%) | 2  (15%) | 13 |
| **Endo-vascular infections** | **23**  **(18%)** | **18**  **(14%)** | **30**  **(23%)** | **14**  **(11%)** | **13**  **(10%)** | **31**  **(24%)** | **129** |
| - Endocarditis | 13 (16%) | 14  (18%) | 17 (21%) | 10  (13%) | 8  (10%) | 18 (21%) | 80 |
| - Native valve endocarditis | 10  (18%) | 9  (16%) | 14  (25%) | 5  (9%) | 7  (13%) | 10  (18%) | 55 |
| - Prosthetic valve endocarditis | 3  (12%) | 5  (20%) | 3  (12%) | 5  (20%) | 1  (4%) | 8  (32%) | 25 |
| - Pacemaker infection | 2 (14%) | 0 | 5 (36%) | 0 | 3  (21%) | 4  (29%) | 14 |
| - Vascular implant infections | 5 (21%) | 2  (8%) | 5  (21%) | 1  (4%) | 2  (8%) | 9  (38%) | 24 |
| - Septic thrombophlebitis | 0 | 0 | 2  (40%) | 1  (20%) | 2  (40%) | 0 | 5 |
| - Other | 4  (29%) | 3  (21%) | 2  (14%) | 2  (14%) | 1  (7%) | 2  (14%) | 14 |
| **Intra-abdominal infections** | **16 (14%)** | **15 (13%)** | **14 (12%)** | **23 (20%)** | **28 (25%)** | **17 (15%)** | **113** |
| - Hepatic abscess | 7  (21%) | 8  (24%) | 2  (6%) | 9  (26%) | 5  (15%) | 3  (9%) | 34 |
| - Intraabdominal abcess | 3  (10%) | 3  (10%) | 4  (13%) | 6  (20%) | 8  (27%) | 6  (20%) | 30 |
| - Peritonitis | 4  (21%) | 1  (5%) | 1  (5%) | 3  (16%) | 8  (42%) | 2  (11%) | 20 |
| - Biliary tract infections | 2  (13%) | 3  (20%) | 1  (7%) | 4  (27%) | 4 (27%) | 1  (7%) | 15 |
| - Colitis/anal and peri-sigmoid abcess | 1  (33%) | 0 | 1 (33%) | 1 (33%) | 0 | 0 | 3 |
| - Other | 4  (9%) | 3  (7%) | 5  (11%) | 10 (23%) | 14 (32%) | 8  (18%) | 44 |
| **Urinary tract infections** | **13**  **(16%)** | **16**  **(19%)** | **17 (20%)** | **9**  **(11%)** | **17 (20%)** | **12 (14%)** | **84** |
| **Intrathoracic infections** | 11  (16%) | 13  (19%) | 11 (16%) | 10  (14%) | 7  (10%) | 17 (25%) | **69** |
| - Empyema | 2  (13%) | 0 | 0 | 6  (40%) | 0 | 7  (47%) | 15 |
| - Pulmonary abscess | 2  (33%) | 4  (67%) | 0 | 0 | 0 | 0 | 6 |
| **Others*** | 27  (23%) | 20  (17%) | 14 (12%) | 12  (10%) | 22 (19%) | 22 (19%) | **117** |
| **Total** | **121**  **(16%)** | **124**  **(17%)** | **122**  **(16%)** | **106**  **(14%)** | **133**  **(18%)** | **143**  **(19%)** | **749** |

**Table 2. Antibiotics used for OPAT of** ≥**14 days**

| **Antibiotic** | **2017** | **2018** | **2019** | **2020** | **2021** | **2022** | **Total** |
| --- | --- | --- | --- | --- | --- | --- | --- |
| Penicillin | 0 | 0 | 6 | 4 | 6 | 12 | 28 |
|  |  |  | 4.8% | 3.8% | 4.5% | 8.4% |  |
| Amoxicillin | 6 | 6 | 9 | 3 | 9 | 9 | 42 |
|  | 5% | 4.9% | 7.3% | 2.8% | 6.8% | 6.3% |  |
| Flucloxacillin | 24 | 25 | 18 | 17 | 17 | 14 | 115 |
|  | 20.0% | 20.3% | 14.5% | 16.0% | 12.8% | 9.8% |  |
| Cefazolin | 5 | 3 | 1 | 3 | 9 | 4 | 25 |
|  | 4.2% | 2.4% | 0.8% | 2.8% | 6.8% | 2.8% |  |
| Ceftriaxone | 33 | 27 | 29 | 19 | 18 | 28 | 154 |
|  | 27.5% | 22.0% | 23.4% | 17.9% | 13.5% | 19.6% |  |
| Cefepime | 8 | 5 | 7 | 6 | 9 | 16 | 51 |
|  | 6.7% | 4.1% | 5.7% | 5.7% | 6.8% | 11.2% |  |
| Ertapenem | 14 | 13 | 16 | 11 | 21 | 13 | 88 |
|  | 11.7% | 10.6% | 12.9% | 10.4% | 15.8% | 9.1% |  |
| Meropenem | 3 | 6 | 3 | 7 | 4 | 3 | 26 |
|  | 2.5% | 4.9% | 2.4% | 6.6% | 3.0% | 2.1% |  |
| Vancomycin | 10 | 12 | 15 | 19 | 13 | 14 | 83 |
|  | 8.3% | 9.8% | 12.1% | 17.9% | 9.8% | 9.8% |  |
| Piperacillin- Tazobactam | 11  9.2% | 19  15.5% | 12  9.7% | 10  9.4% | 16  12.0% | 11  7.7% | 79 |
| Daptomycin | 1 | 2 | 5 | 0 | 2 | 7 | 17 |
|  | 0.8% | 1.6% | 4.0% |  | 1.5% | 4.9% |  |
| Other | 5 | 5 | 3 | 7 | 9 | 12 | 41 |
|  | 4.2% | 4.1% | 2.4% | 6.7% | 6.8% | 8.3% |  |
| Total | 120 | 123 | 124 | 106 | 133 | 143 | 749 |
|  |  |  |  |  |  |  |  |
